# Overnight Dynamics of Ventricular Cerebrospinal Fluid Amyloid‐Beta, Lactate and Hypocretin in Patients With Hydrocephalus: A Pilot Study

**DOI:** 10.1111/jsr.70292

**Published:** 2026-01-29

**Authors:** Casper Schwartz Riedel, Joachim Birch Milan, Niklas Rye Jørgensen, Poul Jennum, Marianne Juhler

**Affiliations:** ^1^ Department of Neurosurgery, Centre of Neuroscience Copenhagen University Hospital—Rigshospitalet Copenhagen Denmark; ^2^ Department of Clinical Neurophysiology, Danish Center for Sleep Medicine Copenhagen University Hospital—Rigshospitalet Glostrup Denmark; ^3^ Centre for Functional and Diagnostic Imaging and Research Copenhagen University Hospital Amager and Hvidovre Hvidovre Denmark; ^4^ Department of Clinical Chemistry, Centre of Diagnostic Investigation Copenhagen University Hospital—Rigshospitalet Glostrup Denmark; ^5^ Department of Clinical Medicine University of Copenhagen Copenhagen Denmark

**Keywords:** Alzheimer's disease, Aβ42, circadian, glymphatic, orexin, sleep

## Abstract

This pilot observational study evaluated whether frequent overnight sampling of ventricular cerebrospinal fluid could clarify how sleep, hypocretin and lactate relate to amyloid‐β42 dynamics in adults with hydrocephalus. Seven participants underwent hourly ventricular cerebrospinal fluid sampling from early evening to late morning during inpatient monitoring, combined with full polysomnography. Concentrations of amyloid‐β42, hypocretin, lactate, melatonin and electrolytes were measured and normalised to each individual's mean. Relationships with sleep stage and circadian patterns were examined using correlation analysis and cosinor modelling. Sleep was markedly disrupted, with obstructive sleep apnea common and analysable sleep data available for six participants. Non‐rapid eye movement sleep peaked at approximately 4 AM Amyloid‐β42 rose in the evening, plateaued during peak non‐rapid eye movement sleep and increased sharply after 8 AM Hypocretin and lactate were positively correlated and each preceded and correlated with amyloid‐β42 surges. Melatonin peaked near 6 AM and was associated with non‐rapid eye movement sleep. Intracranial pressure displayed a strong circadian rhythm, peaking during non‐rapid eye movement sleep, whereas hypocretin and amyloid‐β42 exhibited only modest rhythmicity. These findings demonstrate that overnight ventricular cerebrospinal fluid sampling is feasible in adults with hydrocephalus. Preliminary evidence suggests that processes linked to wakefulness, rather than sleep or intrinsic circadian timing, may be the primary drivers of overnight amyloid‐β42 variation. Hypocretin pathways may represent potential therapeutic targets in Alzheimer's disease, but conclusions are limited by abnormal sleep architecture and underlying neurological disease. Validation in larger and more representative populations is warranted.

## 1 Introduction

Sleep and circadian rhythms are essential for healthy brain function and modulate key processes such as metabolism, hormone secretion and neural plasticity (Tononi and Cirelli 2014; Sharma and Kavuru 2010). Regulation of amyloid‐beta (Aβ) peptides, especially Aβ42, by these biological rhythms has been implicated in the pathogenesis of Alzheimer's disease (AD) (Holth et al. 2019; Alzheimer's Association 2024; Hampel et al. 2021). Both animal and human studies indicate that Aβ42 levels fluctuate with the sleep–wake cycle (Kang et al. 2009; Bateman et al. 2007; Huang, Potter, Sigurdson, Kasten, et al. 2012; Lucey, Mawuenyega, et al. 2017), and that wakefulness and sleep deprivation can lead to increased Aβ42 accumulation in the brain and cerebrospinal fluid (CSF) (Ooms et al. 2014). This suggests disrupted sleep may promote AD pathology, although the exact mechanisms remain unclear (Lucey, Hicks, et al. 2017; Musiek et al. 2015).

Beyond sleep, circadian factors and their disturbance are also linked to neurodegeneration and may influence Aβ dynamics (Musiek et al. 2015; Kress et al. 2018), and melatonin, an established marker of circadian phase, reflects these rhythms (Zisapel 2018). In addition, the neuropeptide hypocretin (orexin), a key regulator of wakefulness and arousal (De Luca et al. 2022; De Lecea and Sutcliffe 2005; Mahoney et al. 2019), has been shown in animal models to influence Aβ accumulation, but its role in human Aβ regulation remains incompletely understood (Kang et al. 2009).

Despite these insights, most human studies rely on lumbar CSF sampling, which may not accurately reflect dynamic changes occurring closer to the brain. Furthermore, the interaction between sleep architecture, ventricular Aβ42 rhythms and arousal‐related biomarkers like hypocretin and lactate in people with disrupted CSF physiology remains poorly characterised.

In this pilot study, we explored the feasibility of frequent overnight ventricular CSF sampling in patients with hydrocephalus to provide preliminary insights into the relationship among sleep stages, Aβ42, hypocretin and lactate fluctuations. Our goal was to identify methodological challenges and guide future studies of sleep‐biomarker interactions in humans.

## 2 Methods

This prospective, observational pilot study was conducted and reported in accordance with the STROBE guidelines for cohort studies (von Elm et al. 2007).

### 2.1 Design

During hospital admission, patients were asked to participate in an additional voluntary sleep evaluation study. This study was conducted alongside intracranial pressure (ICP) monitoring or after endoscopic third ventriculostomy (ETV), with CSF samples collected hourly from the lateral ventricle using a ventricular or ICP catheter. The sampling period lasted from 6 PM to 10 AM on the first night after the ventricular or ICP catheter insertion.

### 2.2 Study Population

Seven patients with suspected hydrocephalus referred to the Copenhagen Neurosurgical Department were enrolled. Each underwent standardised clinical and radiological assessment using the ASPECT Hydrocephalus System and was classified as idiopathic normal‐pressure hydrocephalus (iNPH) or adult‐onset obstructive hydrocephalus (Milan et al. 2022; Toft et al. 2024). iNPH required the classic symptom triad and supportive imaging, with no tumours, subarachnoid haemorrhage, head injury, meningitis or radiological CSF‐flow obstruction. Obstructive hydrocephalus was diagnosed by imaging evidence of CSF pathway blockage. Cohort demographics are summarised in Table 1, and the detailed information for each patient is provided in Table S1.

**TABLE 1:** Demographics and sleep parameters.

	Demographics
Age, years (SD)	63 (12)
Gender	3 male/4 female
Hydrocephalus	1 without hydrocephalus, 2 iNPH and 4 obstructive hydrocephalus
Procedure	3 diagnostic ICP, and 4 ETV
Smoking	2 yes, 2 never and 3 previous
Height, cm (SD)	170 (9)
Weight, kg (SD)	80 (19)
BMI, kg/m^2^ (SD)	27 (5)
Cognitive impairment	3 yes, 4 no

	Sleep parameters
Total sleep time, min (SD)	260 (150)
Percentage of N1 (SD)	36 (22)
Percentage of N2 (SD)	52 (13)
Percentage of N3 (SD)	9 (15)
Percentage of REM (SD)	3 (6)
Arousals (SD)	125 (56)
Apnea hypopnea index (SD)	30 (17)
Epworth sleepiness scale (SD)	8 (4)

Abbreviations: ETV, endoscopic third ventriculostomy; ICP, intracranial pressure monitoring; iNPH, idiopathic normal pressure hydrocephalus; SD, standard deviation.

### 2.3 Ventricular Catheters and ICP Monitoring

NEUROVENT tip‐transducer catheters (RAUMEDIC) were inserted into the right lateral ventricle of three patients undergoing diagnostic ICP monitoring and one post‐ETV patient; for the remaining three ETV patients, a ventricular catheter was inserted for sampling purposes only. All catheters were removed the following day. For transducer‐equipped cases, ICP was recorded at 100 Hz and reported as hourly means (e.g., the 6 PM value is the average from 5–6 PM).

### 2.4 Polysomnography

Sleep evaluation was performed during in‐hospital diagnostics and related to ICP measurements using a previously described methodology (Riedel et al. 2021). Briefly, the ICP signal (RAUMEDIC AG, Helmbrechts, Germany) was transferred into the polysomnography (PSG) system (SOMNOmedics GmbH, Germany) for precise time‐locking. PSG followed AASM standards and was manually scored by an independent rater under neurophysiologist supervision, both blinded to patient diagnoses.

### 2.5 Cerebrospinal Fluid Sampling

From 6 PM to 10 AM, CSF was withdrawn hourly (3.5 mL) via the ventricular catheter. The first 1.8 mL (dead space) was discarded, leaving 1.7 mL for analysis. Immediately, 200 μL was analysed for lactate on an ABL800 FLEX (Radiometer); the remaining 1.5 mL was placed in polypropylene tubes, centrifuged (2000 rpm, 4°C, 10 min) and aliquoted (5 × 250 μL). One aliquot was kept at 4°C for next‐day electrolyte assays (Na, K, Cl, Mg, Ca) on a Roche Cobas using direct ion‐selective electrodes; the others were frozen at −20°C and transferred to −80°C after the final collection. Each sample represented < 0.7% of daily CSF production and did not affect mean ICP.

### 2.6 Cerebrospinal Fluid Analysis of Aβ, Hypocretin and Melatonin Concentrations

For all three analytes, each sample was assessed in duplicate. All samples were measured in the same assay batch to avoid inter‐plate variation.

Amyloid‐beta 1–42 was measured in CSF using the INNOTEST β‐amyloid (1–42) assay (Fujirebio, Gothenburg, Sweden), which is an enzyme‐linked immunosorbent assay (ELISA) assay. Measurements were performed according to the manufacturer's instructions. Intermediate precision for the assay was determined using internal controls (patient pools) and was < 10% at all three levels (324, 725 and 1539 pg/mL).

Hypocretin was determined in the CSF samples using the orexin A assay (Phoenix Pharmaceuticals, CA, USA), a radioimmunoassay (RIA). Measurements were performed according to the manufacturer's instructions. Intermediate precision was determined using internal controls (patient pools) and was consistently < 15% at all three control levels (51, 183 and 422 ng/L).

Melatonin was measured in the CSF using the Melatonin Direct Saliva ELISA kit (IBL International, Hamburg, Germany) according to the manufacturer's instructions. The intermediary precision was determined using internal controls (patient pools) and was 20% at 2.7 ng/L and 17% at 27 ng/L.

### 2.7 Post‐Processing of Data

Biomarker values were normalised to each patient's mean and expressed as percent deviation. Sleep stages were scored in 30‐s epochs, binned hourly in MATLAB (R2020b), and reported as % wake, NREM and REM. Normalised biomarker levels were then paired with the sleep profile of the preceding hour for subsequent analyses.

### 2.8 Statistical Analysis

Data are reported as mean ± SD (range) or *n* (%). Linear associations were tested with two‐tailed Pearson correlations; *p* < 0.05 denoted significance. All analyses were performed in *R* (v 2023.12.0).

### 2.9 Cosinor Analysis

The raw data were analysed for a circadian rhythm using the methods for cosinor‐rythmometry (Cornelissen 2014). The 24‐h rhythms were characterised by the rhythm parameters: mesor (rhythm adjusted average about which oscillation occurs), amplitude (half the difference between the highest and lowest values of the fitted cosinor curve) and time of peak.

## 3 Results

### 3.1 Patients and Procedure

The patient demographics are shown in Table 1. One patient was suspected of having iNPH but did not meet the diagnostic criteria and was thus classified as having no hydrocephalus. There were no complications, and all patients were discharged from the hospital after the completion of the study procedures.

### 3.2 Sleep

**FIGURE 1:**
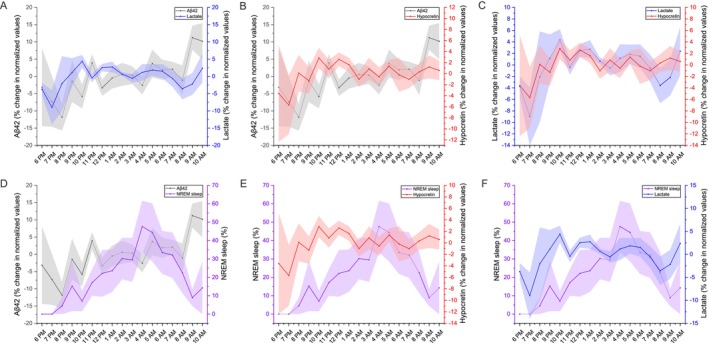
Normalised mean overnight profiles of ventricular Aβ42, lactate, hypocretin and NREM sleep from 6 PM to 10 AM. Mean normalised concentrations of Aβ42, lactate and hypocretin (*n* = 7), and percentage of time spent in NREM sleep (*n* = 6), are plotted from 6 PM to 10 AM shaded areas represent the standard error of the mean (SEM). Aβ42 levels declined to a nadir at 8 PM (−11.8%), then rose and stabilised between midnight and 8 AM, coinciding with peak NREM sleep. A secondary rise in Aβ42 was observed between 9 and 10 AM both hypocretin and lactate reached their lowest values at 7 PM, preceding the nadir of Aβ42 by about 1 h. Afterward, hypocretin and lactate increased, stabilising near their respective mean values from 2 AM onward.

The sleep study revealed that unusual sleep conditions due to the study equipment, sampling procedures and study setting led to poor sleep quality in all participants, leading to fragmented sleep. Despite this, six out of seven participants still achieved sufficient sleep quality for analysing sleep stages. One participant's sleep could not be objectively assessed because of displacement of the central reference electrode; however, the patient was observed sleeping during CSF collection. Consequently, the patient was excluded from NREM sleep analysis (Patient 3). Another patient reported being mostly awake, which was confirmed by PSG sleep data (Patient 5). NREM sleep was recorded in five participants, with four entering deep NREM3 sleep, although briefly in two of them. REM sleep was recorded in two participants. Among the seven patients, five had moderate‐to‐severe obstructive sleep apnea (OSA), one exhibited normal respiration, while respiration patterns could not be evaluated in Patient 3 due to insufficient data. A comparison of all participants (*n* = 6, excluding Patient 3) with normalised values showed that NREM sleep peaked at 4 AM (Figure 1D–F). Patient characteristics are summarised in Table 1, and detailed data for each participant are shown in Table S1.

### 3.3 Biomarkers

The means and ranges of all the biomarkers are shown in Table 2.

**TABLE 2:** Biomaker mean and individual patient range.

Biomaker	*n*	Mean (±SD)	Individual range
Aβ42	7	614 pg/mL (±174)	395–938 pg/mL
Hypocretin	7	623 pmol/L (±102)	436–771 pmol/L
Lactate	7	2.2 mmol/L (±0.6)	1.5–3.3 mmol/L
Melatonin	7	Peak: 92 pg/mL	5.4–246 pg/mL
ICP	4	4.0 mmHg (±3.4)	0.1–8.3 mmHg

### 3.4 Aβ42

**FIGURE 2:**
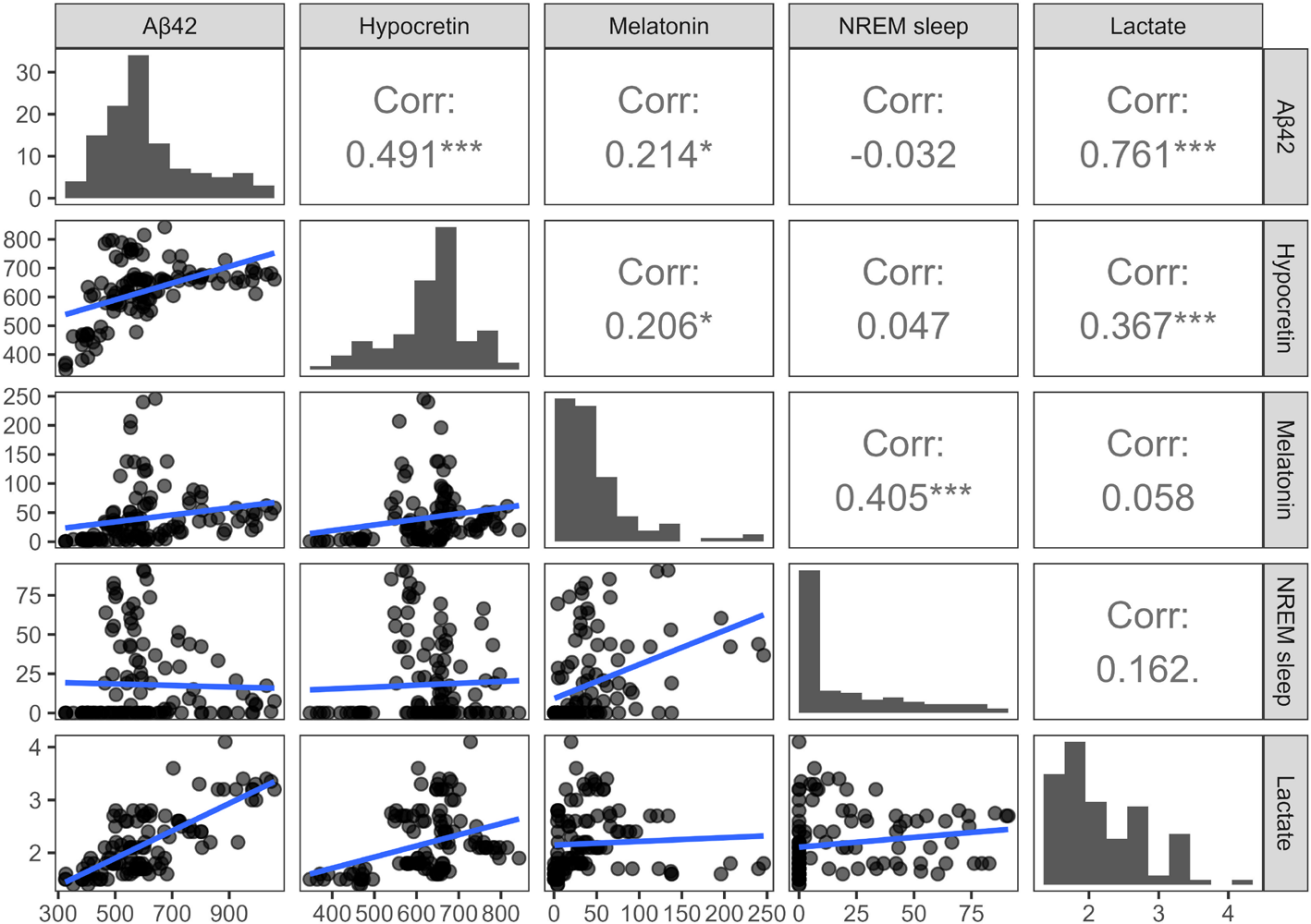
Correlation matrix and scatter plots for Aβ42, hypocretin, lactate, melatonin and NREM sleep. Pairwise Pearson's correlations among amyloid‐β42 (Aβ42), hypocretin, lactate, melatonin and NREM sleep are depicted for the combined patient cohort. The lower triangle shows scatter plots for each variable pair with superimposed linear regression lines, while the upper triangle displays Pearson's r correlation coefficients. Diagonal panels illustrate the distribution of each variable. Aβ42 showed a strong positive correlation with lactate (*r* = 0.76, *p* < 0.001) and a moderate correlation with hypocretin (*r* = 0.49, *p* < 0.001). Lactate and hypocretin were also significantly correlated (*r* = 0.37, *p* < 0.001). Melatonin and NREM sleep correlated moderately (*r* = 0.41, *p* < 0.001). Other pairs showed weaker or non‐significant associations.

Ventricular Aβ42 declined to a nadir of −11.8% at 8 PM, rose to +3.9% at 11 PM and then remained stable between midnight and 8 AM, coinciding with peak NREM sleep (Figure 1A,B,D). A secondary rise was observed between 9 and 10 AM. Because lactate and hypocretin reached their minima 1 h earlier (7 PM), their fluctuations appear to precede those of Aβ42. Aβ42 correlated closely with lactate (*r* = 0.761, *p* < 0.001) and with hypocretin (*r* = 0.491, *p* < 0.001) (Figure 2).

### 3.5 Hypocretin

Hypocretin varied markedly between 6 PM and 8 PM, reaching its lowest value at 7 PM (−5.7%), followed by a rise to +2.8% at 10 PM and stabilisation near the mean from 2 AM to 10 AM (Figure 1B,C,E).

### 3.6 Lactate

Lactate mirrored the hypocretin profile, falling to −9.0% at 7 PM and increasing to +4.4% by 10 PM, after which it stabilised through 1 AM to 10 AM (Figure 1A,C,F). Lactate and hypocretin were significantly correlated (*r* = 0.367, *p* < 0.001) (Figure 2).

### 3.7 Melatonin

The timing of melatonin peaks in the patients varied between 11 PM and 7 AM In four patients, melatonin peaked between 5 AM and 6 AM; across all patients, normalised melatonin values peaked around 6 AM (Table S1), closely tracking the NREM pattern (Figure S1). This is supported by a highly significant correlation (*r* = 0.405, *p* < 0.001) between Melatonin and NREM sleep (Figure 2).

### 3.8 ICP

Mean ICP values remained near zero or slightly negative in the afternoon and morning but peaked around the time of maximal NREM sleep (Figures S2 and S4).

### 3.9 Electrolytes

The normalised levels of all electrolytes showed minimal variation, which remained within ±1% throughout the study period (Figure S3).

### 3.10 Cosinor Analysis

**FIGURE 3:**
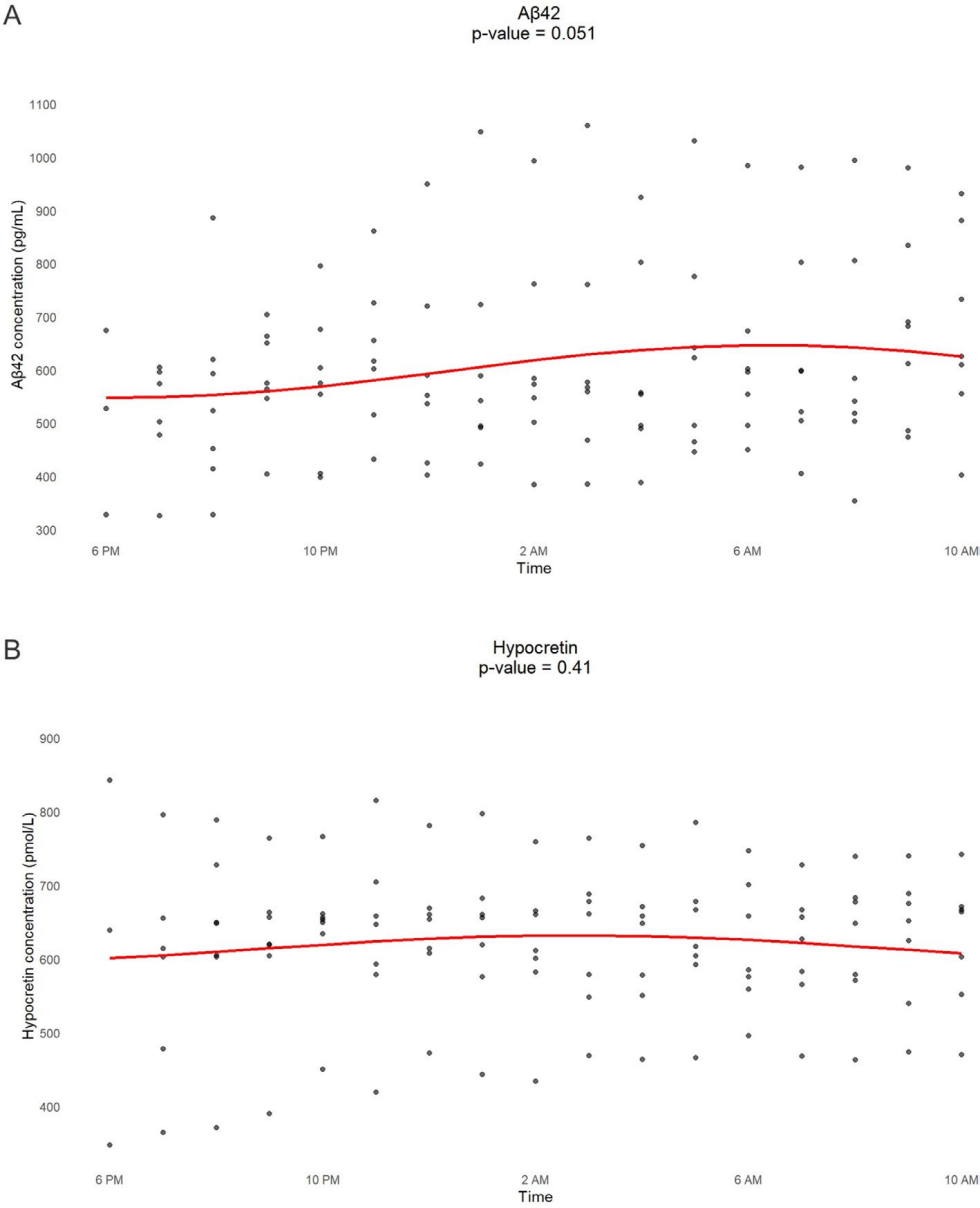
Cosinor analysis of circadian rhythms in ventricular Aβ42 and hypocretin. Cosinor analysis was performed to assess underlying circadian rhythmicity in (A) Aβ42 and (B) hypocretin concentrations across all patients (*n* = 7). Each black dot represents a raw biomarker value, and the red line displays the fitted cosinor curve. Aβ42 demonstrated a small but significant circadian variation, with a relative amplitude of 8%. In contrast, hypocretin showed no significant circadian rhythm, with a relative amplitude of only 3%.

Finally, we conducted a cosinor analysis to investigate the circadian rhythms of Aβ42, hypocretin and ICP. The analysis revealed that Aβ42 exhibited small but significant circadian variation, with a relative amplitude of 8% (Figure 3A). However, hypocretin did not display a significant circadian rhythm, with a relative amplitude of only 3% (Figure 3B). In contrast, ICP demonstrated significant circadian rhythm variation, with a substantial relative amplitude of 259%. Notably, the average ICP values were close to zero or even negative in the afternoon and morning and peaked around the peak in NREM sleep (Figures S2 and S4 and Table 3).

**TABLE 3:** Diurnal rhythm of Aβ42, hypocretin and ICP.

	Unit	Mesor (SD)	Output from cosinor analyses			
			Amplitude	Peak, clock time	Cosinor *p*	Relative amplitude %
Aβ42	pg/mL	597.2 (20.3)	49.3	06:20 AM	0.05	8
Hypocretin	pmol/L	613.6 (12.2)	18.6	02:47 AM	0.41	3
ICP	mmHg	2.1 (0.6)	5.4	03:20 AM	0.001	259

*Note:* Data were analysed using cosinor analysis, and Aβ42 and hypocretin levels did not follow a significant diurnal 24‐h rhythm (*n* = 7). Amplitude: half the difference between the highest and lowest values of the fitted cosinor curve; relative amplitude: 100 × (amplitude/mesor).

## 4 Discussion

This pilot study is the first to directly assess overnight levels of Aβ42, hypocretin and lactate in ventricular CSF, offering new insight into the rapid dynamics of brain‐derived biomarkers. By sampling at the ventricular level, our study minimises the dilution and delay seen with lumbar CSF analyses, providing a more immediate window into brain physiology.

### 4.1 Arousal, Hypocretin and Aβ42

Our main finding is that ventricular Aβ42 fluctuations are tightly linked to changes in arousal markers, hypocretin and lactate, with increases in these markers preceding rises in Aβ42. In contrast, while periods of maximal NREM sleep were associated with lower Aβ42 levels, this effect was modest and not statistically significant. The observed close relationship between hypocretin and lactate further highlights the role of arousal‐related biology, since both markers increase during wakefulness and reflect heightened neuronal activity (Lucey, Hicks, et al. 2017; Naylor et al. 2012; Blouin et al. 2013).

### 4.2 Comparison With Lumbar CSF Sampling Studies

Prior studies using continuous lumbar CSF collection in healthy adults and patients with Alzheimer's disease have consistently reported robust circadian rhythms in Aβ42 (with up to 28% amplitude) and clear daily rhythmicity in hypocretin (Slats et al. 2013; Slats, Claassen, Lammers, et al. 2012; Spies et al. 2011; Slats, Claassen, Spies, et al. 2012; Lucey et al. 2015). In our ventricular samples, Aβ42 exhibited only subtle circadian fluctuations (~8% amplitude) while hypocretin showed minimal rhythmicity. These differences may relate to site‐specific factors, including a more immediate origin of ventricular CSF and altered CSF dynamics in our hydrocephalus cohort, as well as the higher temporal resolution in our study. Importantly, our data suggest that in the ventricular compartment, arousal‐related signals provide a more immediate and dominant influence on Aβ42 than sleep stage or circadian processes.

### 4.3 Wakefulness as the Principal Driver

The strong temporal coupling of Aβ42 with both hypocretin and lactate, and the weaker link of Aβ42 with NREM sleep, support the idea that wakefulness‐related neuronal activity is the primary regulator of Aβ dynamics in this context. These findings build on previous evidence from animal studies (Kang et al. 2009) and lumbar CSF analyses (Bateman et al. 2006) but provide new human evidence that production or release of Aβ42 into CSF is likely more strongly coupled to arousal than to sleep‐related clearance.

### 4.4 Therapeutic Implications

Mounting evidence, including ours, suggests that interventions targeting the hypocretin/orexin system may reduce Aβ accumulation (Kang et al. 2009; Herring et al. 2020). Pharmacologic reduction of hypocretin signalling with dual orexin receptor antagonists has been shown to dampen Aβ levels in animal models; our findings in human ventricular CSF further support the potential therapeutic relevance of hypocretin pathway modulation in Alzheimer's disease.

### 4.5 Novelty of Hypocretin‐Lactate Coupling

A distinctive aspect of our study is the observed correlation between hypocretin and lactate, two arousal‐associated markers (Lucey, Hicks, et al. 2017; Naylor et al. 2012; Blouin et al. 2013). Lactate dynamics have been closely tied to neuronal activity and sleep‐wake state, and this finding supports an integrated arousal/wakefulness mechanism influencing both metabolic and neuropeptide signals that regulate Aβ homeostasis.

### 4.6 Circadian Rhythmicity in ICP

Finally, we identified a pronounced circadian rhythm in ICP, peaking during NREM sleep, suggesting an additional component of sleep–wake or circadian regulation of CSF physiology, independent of postural effects.

## 5 Methodological Considerations and Study Limitations

This study should be viewed as an exploratory, pilot investigation with important methodological limitations. The small sample size and focus on patients with hydrocephalus or suspected hydrocephalus restrict the generalisability of our findings. All but one participant had significant CSF flow abnormalities, and many also had comorbidities such as obstructive sleep apnea, commonly observed in individuals with iNPH (Riedel et al. 2022), and recent neurosurgery, both of which are known to independently affect sleep architecture, ICP and CSF biomarker dynamics (Riedel et al. 2021; Riedel et al. 2023; Bu et al. 2015; Ju et al. 2019; Kim et al. 2019). Furthermore, most participants exhibited Aβ42 concentrations below established diagnostic thresholds, raising the possibility of underlying neurodegenerative disease in this clinical cohort; this means our results may reflect disease‐specific rather than normal physiological processes. Additional biomarker data such as tau and phosphorylated tau were not collected, and thus finer phenotyping with respect to underlying pathology was not possible.

Our study design was also limited using a single night of sleep monitoring in a hospital environment. The first‐night effect (Agnew et al. 1967; Tamaki et al. 2016), fragmented and disrupted sleep, very limited REM and N3 sleep, and frequent obstructive sleep apnea events in this setting complicate interpretation of sleep stage effects on biomarker kinetics. As a result, the relationships observed between sleep stages and CSF Aβ42 dynamics should be interpreted with caution and may not be representative of either healthy physiology or general patient populations.

In addition, as our analysis was constrained to a single overnight sampling period, we could not fully distinguish true diurnal or sleep‐related fluctuations from potential monotonic increases in analyte levels due to technical or procedural artefacts, a phenomenon well‐documented in serial lumbar CSF sampling studies. Although we employed materials designed to minimise Aβ42 aggregation (Del Campo et al. 2012) and our findings of a linear rise in Aβ42 levels align with prior reports (Lucey, Mawuenyega, et al. 2017; Huang, Potter, Sigurdson, Santacruz, et al. 2012), the possibility of sampling‐related artefacts cannot be excluded. The stability of electrolyte measurements (±1% variation) throughout the study supports the technical reliability of our approach (Figure S3). However, without multi‐day sampling, it is not possible to definitively separate sampling drift from biologically meaningful changes.

Finally, while direct ventricular sampling provides superior temporal resolution compared to lumbar CSF studies, the unique patient population and perioperative context in this study limit the generalisability of our findings. Nonetheless, our results highlight the feasibility and potential value of direct ventricular biomarker monitoring for investigating sleep–wake regulation in the human brain. Future research should incorporate larger, more diverse and well‐characterised cohorts, multi‐night sampling, comprehensive biomarker panels, including tau and p‐tau, and address confounders such as obstructive sleep apnea. Additionally, simultaneous sampling from both ventricular and lumbar CSF could elucidate the kinetics and transmission delay of biomarkers, facilitating direct comparison with other studies and enhancing interpretation of results across different sampling sites.

## 6 Conclusion

This pilot study provides the first direct evidence in humans that wakefulness‐related processes, including arousal markers such as hypocretin and lactate, are closely linked to rapid changes in ventricular Aβ42 levels, while the impact of sleep stages such as NREM appears more modest. These findings highlight the potential importance of arousal pathways in amyloid dynamics and support ongoing exploration of hypocretin as a therapeutic target for Alzheimer's disease. However, due to the small, clinically selected hydrocephalus cohort and methodological constraints, these results should be interpreted as preliminary. Future research should validate and extend these observations in larger, more diverse populations, with improved control for comorbidities and sleep quality, to better understand the interplay of sleep, arousal and neurodegenerative disease risk.

## Author Contributions

**Casper Schwartz Riedel:** investigation, conceptualization, funding acquisition, writing – original draft, methodology, visualization, writing – review and editing, formal analysis, project administration, data curation. **Joachim Birch Milan:** conceptualization, investigation, writing – review and editing, methodology. **Niklas Rye Jørgensen:** methodology, writing – review and editing, formal analysis. **Poul Jennum:** conceptualization, funding acquisition, writing – review and editing, methodology, supervision, resources. **Marianne Juhler:** conceptualization, funding acquisition, writing – review and editing, methodology, supervision, resources.

## Funding

This work was supported by the Lundbeck Foundation (R211‐2015‐2937), the Alzheimer's Research Fund, the IMK Almene Fund, the Novo Nordisk Tandem (NNF17OC0024718) and the Danish Cardiovascular Academy (NNF20SA0067242).

## Disclosure

The authors have nothing to report.

## Ethics Statement

The study protocol was approved by the Committee on Health Research Ethics for the Capital Region of Denmark (H‐1‐2014‐123) and was performed in accordance with the Declaration of Helsinki. Written informed consent was obtained from all patients.

## Conflicts of Interest

The authors declare no conflicts of interest.

## Supporting information


**Figure S1:** Normalised mean values from 6 PM to 10 AM (Melatonin, *n* = 7, NREM sleep, *n* = 6). The shaded area is S.E.M.
**Figure S2:** Normalised mean values from 6 PM to 10 AM (ICP, *n* = 4, NREM sleep, *n* = 6). The shaded area is S.E.M.
**Figure S3:** Cosinor analysis of ICP to assess underlying circadian rhythms. Black dots represent raw data points from all patients (*n* = 4), while the red line depicts the fitted cosinor curve.
**Figure S4:** Normalised mean values from 6 PM to 10 AM. The shaded area is S.E.M.
**Table S1:** Patient‐level time‐stamped CSF biomarkers, sleep, lactate and intracranial pressure metrics.

## Data Availability

Anonymised data are available upon reasonable request from the corresponding author and after clearance from a competent ethics committee.
