# Supplementary material for: Overnight Dynamics of Ventricular Cerebrospinal Fluid Amyloid‐Beta, Lactate and Hypocretin in Patients With Hydrocephalus: A Pilot Study
Source: J Sleep Res. 2026 Jan 29;35(4):e70292. doi: 10.1111/jsr.70292 (PMC13357650; doi:10.1111/jsr.70292)
Supplement: Supplementary file 1 — Figure S1: Normalised mean values from 6 PM to 10 AM (Melatonin, n = 7, NREM sleep, n = 6). The shaded area is S.E.M. Figure S2: Normalised mean values from 6 PM to 10 AM (ICP, n = 4, NREM sleep, n = 6). The shaded area is S.E.M. Figure S3: Cosinor analysis of ICP to assess underlying circadian rhythms. Black dots represent raw data points from all patients (n = 4), while the red line depicts the fitted cosinor curve. Figure S4: Normalised mean values from 6 PM to 10 AM. The shaded area is S.E.M. Table S1: Patient‐level time‐stamped CSF biomarkers, sleep, lactate and intracranial pressure metrics. [file JSR-35-e70292-s001.pdf]

Supplementary figures and tables.

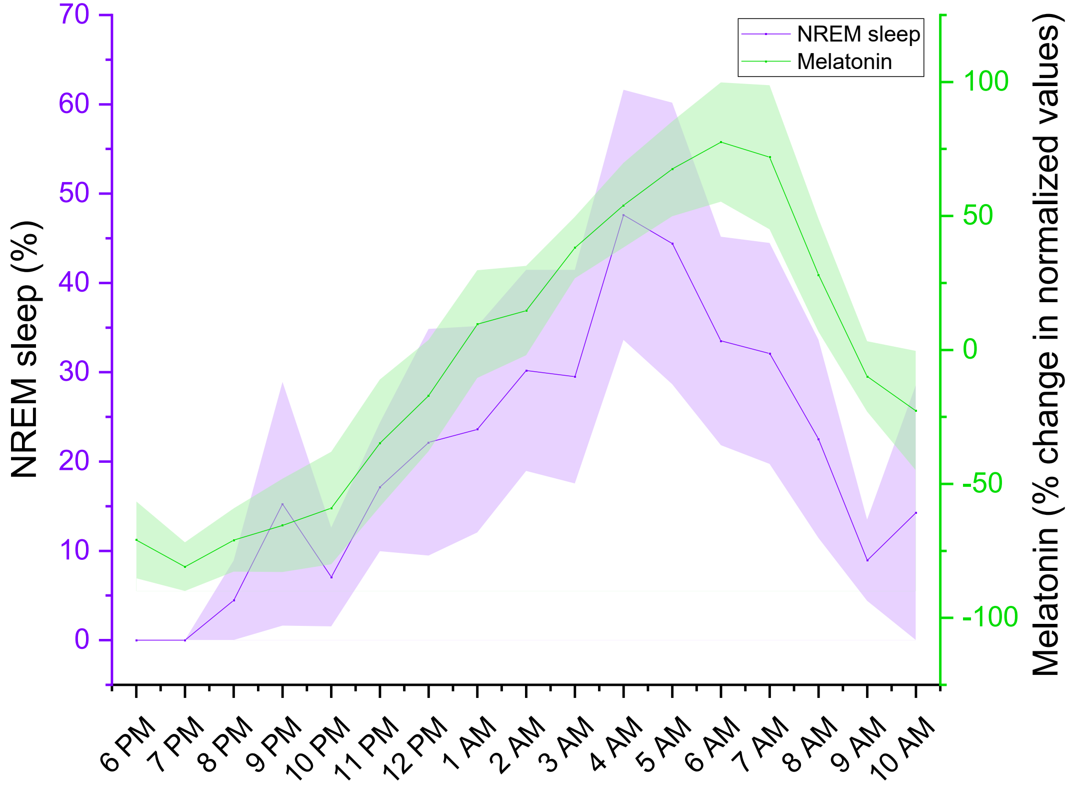

Supplementary figure 1. Normalized mean values from 6 PM to 10 AM (Melatonin, n = 7, NREM sleep, n = 6). The shaded area is S.E.M.

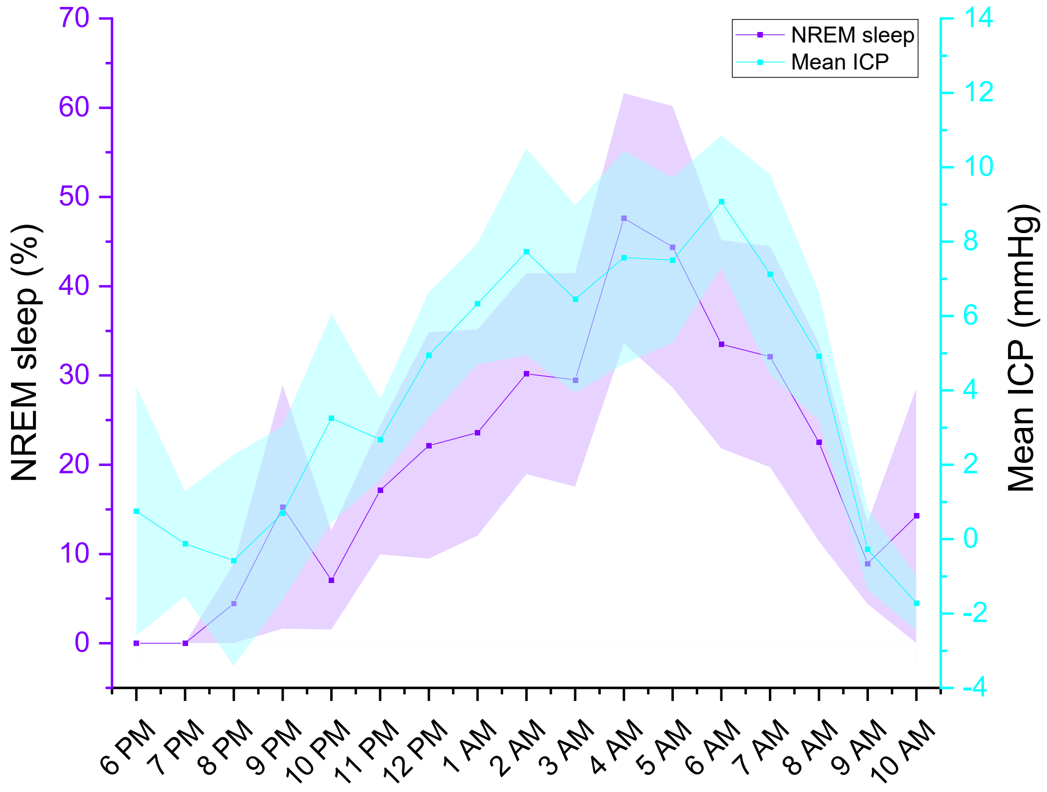

Supplementary figure 2. Normalized mean values from 6 PM to 10 AM (ICP, n = 4, NREM sleep, n = 6). The shaded area is S.E.M.

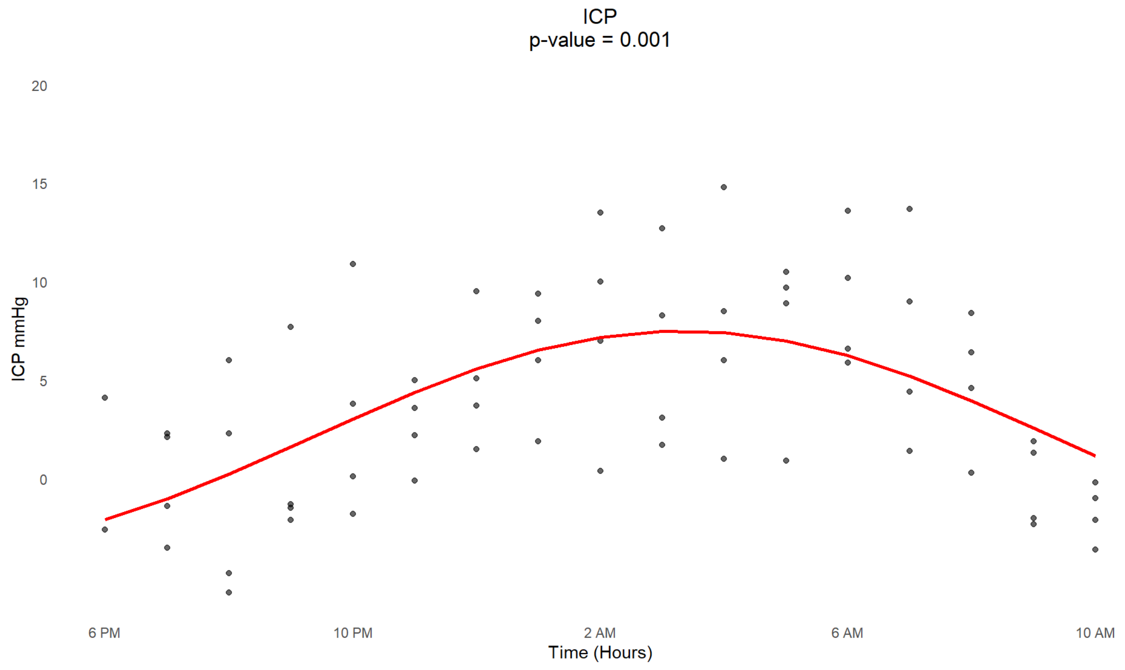

Supplementary Figure 3. Cosinor analysis of ICP to assess underlying circadian rhythms. Black dots represent raw data points from all patients (n=4), while the red line depicts the fitted cosinor curve.

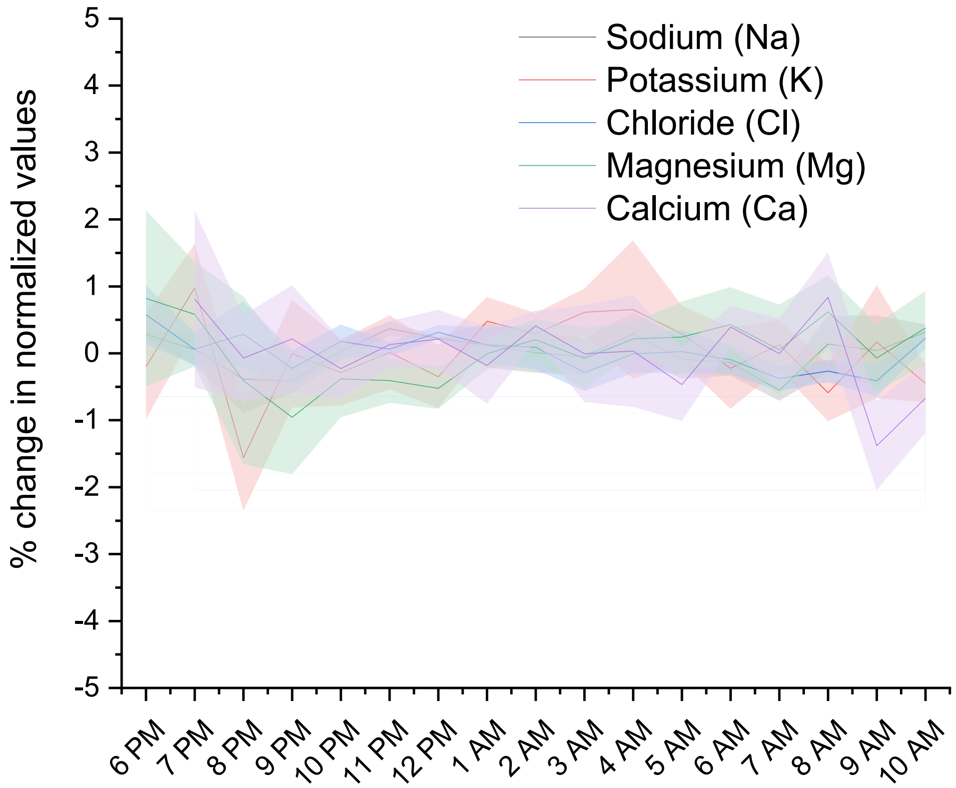

Supplementary figure 4. Normalized mean values from 6 PM to 10 AM. The shaded area is S.E.M.

Supplementary Table 1. Patient-level time-stamped CSF biomarkers, sleep, lactate, and intracranial pressure metrics

Patient_ID	Time	Aβ42	Hypocretin	Melatonin	NREM_sleep	Lactate	icp_mean	icp_med
1	1	502,0	614,0	4,2	0,0			
1	2	593,0	605,0	5,1	22,3			
1	3	564,0	657,0	4,3	69,6	2,8		
1	4	676,0	653,0	4,9	28,6	2,7		
1	5	726,0	704,0	16,0	29,5	2,6		
1	6	720,0	669,0	25,0	46,3	2,6		
1	7	723,0	656,0	37,0	51,3	2,6		
1	8	761,0	660,0	66,0	43,9	2,5		
1	9	760,0	688,0	74,0	9,2	2,4		
1	10	802,0	671,0	86,0	42,3	2,4		
1	11	775,0	667,0	89,0	15,0	2,4		
1	12	673,0	658,0	96,0	2,5	2,4		
1	13	802,0	667,0	76,0	0,0	2,4		
1	14	805,0	677,0	54,0	0,0	2,1		

1	15	834,0	675,0	37,0	0,0	2,2		
1	16	931,0	667,0	35,0	0,0	2,2		
2	0	527,0	639,0	12,0	0,0	1,6		
2	1	574,0	478,0	11,0	0,0	1,7		
2	2	619,0	650,0	9,6	0,0	1,6		
2	3	663,0	663,0	9,9	0,0	1,8		
2	4	575,0	661,0	9,1	0,0	1,8		
2	5	655,0	658,0	14,0	0,0	1,7		
2	6	590,0	660,0	40,0	25,7	1,7		
2	7	589,0	660,0	92,0	13,0	1,7		
2	8	573,0	665,0	75,0	33,7	1,7		
2	9	567,0	678,0	137,0	53,0	1,7		
2	10	554,0	658,0	196,0	60,4	1,6		
2	11	641,0	617,0	246,0	36,8	1,8		
2	12	554,0	559,0	207,0	42,2	1,8		
2	13	598,0	627,0	240,0	43,9	1,7		
2	14	541,0	648,0	138,0	19,0	1,6		
2	15	682,0	652,0	138,0	0,0	1,6		

2	16	609,0	664,0	123,0	0,0	1,7		
3	-1	384,0	380,0	2,4		1,5	3,4	2,6
3	0	327,0	347,0	0,5		1,5	4,1	2,9
3	1	325,0	364,0	0,5		1,5	2,3	1,5
3	2	327,0	371,0	0,5		1,6	6,0	4,6
3	3	404,0	390,0	0,5		1,6	7,7	7,3
3	4	398,0	450,0	0,5		1,6	10,9	10,0
3	5	432,0	419,0	2,8		1,5	5,0	4,0
3	6	402,0	472,0	4,3		1,6	9,5	8,5
3	7	423,0	443,0	5,4		1,6	8,0	6,9
3	8	384,0	434,0	4,4		1,5	13,5	12,0
3	9	385,0	469,0	4,3		1,5	12,7	11,5
3	10	388,0	464,0	5,0		1,7	14,8	13,6
3	11	446,0	466,0	4,5		1,5	10,5	9,6
3	12	450,0	496,0	4,4		1,5	13,6	12,0
3	13	405,0	468,0	4,3		1,4	13,7	11,7
3	14	353,0	463,0	4,0		1,4	8,4	8,0
3	15	473,0	474,0	2,2		1,5	1,3	0,5

3	16	402,0	470,0	0,5		1,5	-1,0	-1,7
4	0	674	843,0	20,0	0,0	1,9	-2,6	-2,8
4	1	477,0	796,0	22,0	0,0	1,9	-3,5	-3,7
4	2	523,0	789,0	27,0	0,0	2,0	-4,8	-5,0
4	3	575,0	764,0	44,0	0,0	2,1	-2,1	-2,5
4	4	554,0	766,0	51,0	0,0	2,1	0,1	-0,2
4	5	602,0	815,0	61,0	0,0	2,1	2,2	2,0
4	6	552,0	781,0	51,0	43,3	2,1	1,5	1,2
4	7	491,0	797,0	49,0	24,5	2,1	1,9	1,6
4	8	547,0	759,0	39,0	66,5	2,2	0,4	0,0
4	9	559,0	764,0	39,0	20,7	2,1	1,7	1,5
4	10	557,0	754,0	31,0	57,1	2,1	1,0	0,6
4	11	464,0	785,0	34,0	19,0	2,1	0,9	0,5
4	12	596,0	747,0	28,0	0,0	2,1	5,9	5,8
4	13	521,0	728,0	30,0	0,0	2,2	1,4	0,5
4	14	503,0	739,0	27,0	11,7	2,2	0,3	-0,6
4	15	690,0	740,0	20,0	2,5	2,3	-2,3	-2,5
4	16	733,0	742,0	17,0	0,0	2,5	-0,2	-0,6

5	2	886,0	728,0	20,0	0,0	4,1		
5	3	704,0	604,0	26,0	6,7	3,6		
5	4	795,0	650,0	35,0	6,7	3,3		
5	5	861,0	647,0	41,0	33,4	3,2		
5	6	949,0	654,0	44,0	12,6	3,4		
5	7	1047,0	682,0	48,0	0,9	3,4		
5	8	993,0	611,0	48,0	5,0	3,3		
5	9	1059,0	661,0	58,0	7,5	3,2		
5	10	924,0	648,0	58,0	20,7	3,2		
5	11	1031,0	678,0	62,0	17,4	3,4		
5	12	984,0	701,0	51,0	0,0	3,2		
5	13	981,0	657,0	37,0	8,3	3,2		
5	14	994,0	683,0	26,0	5,8	3,0		
5	15	980,0	689,0	20,0	0,0	3,0		
5	16	881,0	671,0	14,0	0,0	3,2		
6	1	596,0	603,0	4,8	0,0	1,8	2,1	1,8
6	2	414,0	603,0	4,4	0,0	1,9	2,3	1,7
6	3	650,0	620,0	4,0	0,0	2,0	-1,5	-1,6

6	4	604,0	656,0	4,7	0,0	2,2	3,8	4,3
6	5	616,0	593,0	4,7	0,0	2,4	3,6	3,7
6	6	536,0	614,0	4,0	0,0	2,6	3,7	3,5
6	7	542,0	619,0	9,0	22,8	2,6	6,0	5,0
6	8	501,0	582,0	18,0	74,0	2,5	7,0	6,8
6	9	577,0	548,0	40,0	63,7	2,6	3,1	2,8
6	10	495,0	550,0	51,0	55,4	2,7	6,0	5,6
6	11	622,0	592,0	66,0	73,7	2,7	8,9	8,5
6	12	602,0	576,0	121,0	90,2	2,7	6,6	6,3
6	13	599,0	565,0	134,0	91,0	2,7	4,4	4,1
6	14	518,0	571,0	113,0	42,2	2,7	6,4	6,0
6	15	611,0	540,0	65,0	85,3	2,8	1,9	1,6
6	16	625,0	552,0	76,0	19,0	2,8	-3,6	-3,8
7	1	605,0	655,0	0,5	0,0	1,7	-1,4	-0,8
7	2	452,0	648,0	1,2	0,0	1,6	-5,8	-6,2
7	3	546,0	619,0	0,5	0,0	1,7	-1,3	-3,3
7	4	405,0	634,0	0,5	0,0	1,8	-1,8	-2,0
7	5	515,0	579,0	3,2	0,0	1,8	-0,1	-0,5

7	6	425,0	608,0	12,0	0,0	1,8	5,1	4,3
7	7	494,0	576,0	22,0	0,0	1,8	9,4	8,9
7	8	584,0	601,0	28,0	0,0	1,8	10,0	9,1
7	9	467,0	579,0	31,0	63,9	1,8	8,3	8,0
7	10	489,0	578,0	31,0	52,9	1,8	8,5	8,2
7	11	495,0	604,0	32,0	79,5	1,8	9,7	8,8
7	12	495,0	585,0	37,0	82,7	1,8	10,2	9,2
7	13	504,0	583,0	33,0	76,2	1,8	9,0	8,6
7	14	584,0	579,0	21,0	32,3	1,8	4,6	4,8
7	15	485,0	625,0	28,0	0,0	1,9	-2,0	-2,9
7	16	555,0	603,0	16,0	5,0		-2,1	-2,6

Patient-level dataset with time-stamped measurements of cerebrospinal fluid biomarkers (Aβ42, hypocretin/orexin, melatonin), sleep architecture (NREM sleep), lactate, and intracranial pressure summary metrics (mean and median ICP), indexed by Patient_ID.
